# Differential Impacts of Anthropogenic Disturbance on Community Composition and Alpha Diversity of Macroinvertebrates at a Large Urban River System

**DOI:** 10.1002/ece3.74494

**Published:** 2026-10-22

**Authors:** Huawei Bao, Zongwei Lin, Zhijun Xia, Min Zhang, Xiao Du, Collins Oduro, Sangar Khan, Naicheng Wu

**Affiliations:** ^1^ School of Geography and Remote Sensing, School of Civil Engineering and Future Cities Ningbo University Ningbo China; ^2^ Zhejiang‐Germany Joint Laboratory on Remote Sensing of Coastal Ecosystem Ningbo University Ningbo China; ^3^ Department of Geosciences and Geography University of Helsinki Helsinki Finland; ^4^ State Key Laboratory of Water Cycle and Water Security China Institute of Water Resources and Hydropower Research Beijing China; ^5^ State Key Laboratory of Freshwater Ecology and Biotechnology—Ningbo Laboratory Ningbo China

**Keywords:** anthropogenic activity, functional diversity, functional feeding groups, land use, macroinvertebrate, taxonomic diversity

## Abstract

Macroinvertebrates play critical roles in aquatic ecosystems, yet the effects of anthropogenic land‐use disturbance on macroinvertebrate communities and their taxonomic and functional diversity remain poorly understood, particularly in rapidly urbanizing regions of eastern China. Here, we identified 211 macroinvertibrates species from 385 river sites in Ningbo, Zhejiang Province, China. We classified sites as low‐disturbance (LD) or high‐disturbance (HD) based on surrounding land use. This study examined differences in macroinvertebrate community composition, taxonomic and functional diversity, and functional feeding groups (FFGs) between disturbance levels, and further explored the potential direct and indirect effects of land use and environmental factors using structural equation modeling (SEM). Macroinvertebrate community composition differed significantly (*p* = 0.001) between LD and HD sites, with HD sites showing lower taxonomic and functional diversity. The relative abundance of FFGs also shifted with disturbance level, with filterers, gatherers, and predators more abundant at LD sites, whereas scrapers and shredders increased at HD sites. SEM further indicated that agricultural and urban land uses were negatively associated with both taxonomic and functional diversity, while non‐purgeable organic carbon (NPOC) showed a positive association with diversity. These findings demonstrate that anthropogenic land‐use disturbance can restructure macroinvertebrate communities and reduce multiple dimensions of biodiversity, highlighting the importance of integrating both taxonomic and functional information into ecological assessments of human‐dominated river systems.

## Graphical abstract

High‐disturbance reduced taxonomic and functional diversity. Macroinvertebrate communities differ significantly between disturbance groups. Agricultural and urban land use directly reduced diversity. Land use change profoundly impacts macroinvertebrate communities.

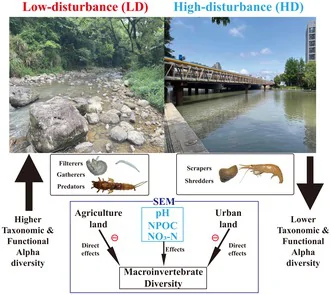

## 1 Introduction

Rivers play a vital role in sustaining freshwater biodiversity and maintaining ecosystem processes and functions (Abell et al. 2008; Vörösmarty et al. 2015). However, freshwater ecosystems are experiencing substantial biodiversity loss worldwide, largely driven by increasing anthropogenic pressures, particularly urbanization and agricultural expansion (Birk et al. 2020; Daskalova et al. 2020; Pillay 2022; Oduro et al. 2025). Changes in surrounding land use can modify hydrological processes, material inputs, habitat structure, and water quality, thereby altering the environmental conditions that regulate aquatic communities. Understanding how land‐use disturbance restructures riverine biological communities is therefore essential for assessing and managing freshwater ecosystems in human‐dominated landscapes.

Urban land uses can affect river ecosystems through partly distinct but interacting pathways. Urban development increases impervious surfaces, pollutant inputs into water bodies, and riparian habitat modification, which can affect water quality and physical habitat complexity (Wang et al. 2012; Liang et al. 2021). In addition, agricultural activities can increase nutrient and sediment delivery through runoff and soil erosion, and pesticide inputs may impose additional chemical stress on aquatic organisms (Rasmussen et al. 2012; Stefanidis et al. 2016; Khan et al. 2025). These land‐use pressures can consequently modify physicochemical conditions, sediment characteristics, habitat availability, and resource environments (Treitler et al. 2016). Such environmental changes may act as ecological filters that favor taxa with specific ecological tolerances and functional traits, ultimately reshaping macroinvertebrate community composition and functional structure (Du et al. 2021). Thus, consider the effects of land use on aquatic biodiversity not only as direct responses to anthropogenic disturbance but also as responses mediated through changes in local environmental conditions.

Biodiversity responses to environmental disturbance can be characterized from both taxonomic and functional perspectives. Taxonomic diversity (TD) describes variation in species richness and composition, whereas functional diversity (FD) reflects the diversity and distribution of ecological traits and functions within communities (Schmera et al. 2017; Kaczmarski et al. 2020). TD and FD may respond differently to environmental change because species turnover does not necessarily produce proportional changes in functional structure (Doni et al. 2026). Functional feeding groups (FFGs) provide an additional ecological perspective by linking community composition to resource use and ecosystem processes. Therefore, integrating taxonomic diversity, functional diversity, and FFG structure can provide a more comprehensive understanding of how anthropogenic land‐use disturbance alters both the composition and ecological roles of macroinvertebrate communities (Craven et al. 2016; Chao et al. 2019).

Macroinvertebrates are widely used as indicators of river ecological condition because of their sensitivity to environmental changes and their close associations with habitat characteristics and water quality (Graeber et al. 2017; Simaika et al. 2024). Land use is a key driver of macroinvertebrate community composition and diversity, but its effects are often scale‐ and context‐dependent. Sponseller et al. (2001) demonstrated that land use and spatial scale jointly influence stream macroinvertebrate communities. Linares et al. (2025) showed that benthic macroinvertebrate assemblages respond to an urbanization gradient in both taxonomic and functional dimensions, indicating that land‐use intensification alters not only species composition but also functional trait structure. Firmiano et al. (2021) further found that land use and local environmental conditions shape macroinvertebrate metacommunity organization in Neotropical stream networks. Therefore, studies have demonstrated that land use can influence macroinvertebrate community composition and diversity.

However, important knowledge gaps remain. First, previous studies (Sponseller et al. 2001; Firmiano et al. 2021; Linares et al. 2025) have often emphasized overall land‐use intensity or individual land‐use categories, whereas how macroinvertebrate communities reorganize across contrasting levels of anthropogenic disturbance remains less well understood. Second, relatively few studies have simultaneously examined taxonomic diversity, functional diversity, and FFG structure to determine whether different dimensions of biodiversity respond consistently to land‐use disturbance. Third, although land use can influence macroinvertebrates through multiple environmental pathways, the relative contributions of direct land‐use effects and indirect effects mediated by local environmental conditions remain poorly resolved, particularly in highly urbanized river networks. Addressing these gaps is important for moving beyond pattern‐based descriptions toward a mechanistic understanding of how land‐use disturbance restructures riverine biodiversity.

Ningbo City, a rapidly urbanizing coastal region in eastern China, provides an appropriate setting for examining these questions because extensive agricultural and urban development has substantially transformed its riverine landscapes (Li et al. 2022; Zhao et al. 2026). Previous studies have reported ecological problems associated with intensified land use in the region, including eutrophication, habitat simplification, and biodiversity loss (Shi et al. 2025). Nevertheless, macroinvertebrate communities' responses to contrasting anthropogenic disturbance levels and the environmental pathways underlying these responses remain unclear. To address this gap, we conducted a large‐scale survey across 385 river sites in Ningbo City. By integrating community composition, taxonomic and functional diversity, FFG structure, environmental variables, and structural equation modeling (SEM), this study provides a multi‐dimensional framework for linking land‐use disturbance with macroinvertebrate biodiversity and its potential environmental mediators. This approach allows us to evaluate not only whether macroinvertebrate communities differ between disturbance levels but also how land‐use and local environmental conditions may jointly contribute to these differences.

Therefore, this study aimed to: (i) characterize differences in macroinvertebrate community composition between low‐ and high‐disturbance river sites; (ii) evaluate the responses of taxonomic diversity, functional diversity, and FFG structure to anthropogenic disturbance; and (iii) identify potential direct and indirect pathways linking agricultural and urban land use, environmental conditions, and macroinvertebrate biodiversity. We hypothesized that: H1, macroinvertebrate community composition would differ significantly between low‐ and high‐disturbance sites because land‐use disturbance alters local habitat and environmental conditions; H2, FFG composition would shift with disturbance level as changes in habitat and resource conditions modify the relative representation of feeding strategies; H3, taxonomic and functional diversity would be lower at high‐disturbance sites because intensified land use increases environmental stress and reduces habitat suitability; and H4, land use would influence macroinvertebrate diversity through both direct pathways and indirect pathways mediated by local environmental conditions.

## 2 Materials and Methods

### 2.1 Study Area

The study area is located in Ningbo City, Zhejiang Province, on the central coast of China and the southern edge of the Yangtze River Delta (120°55′–122°16′ E, 28°51′–30°33′ N). Ningbo has a subtropical monsoon climate with mild temperatures and distinct seasons, and a dense river network that forms a composite ecosystem of inland waterways and coastal rivers.

Over the past two decades, rapid urbanization and socioeconomic growth in the region, reflected by an increase in urbanization rate from 31.9% to 80.9% and an approximately eightfold rise in GDP (http://www.ningbo.gov.cn/), have intensified human activities and landscape modification, thereby exerting increasing pressure on riverine ecosystems (Zhang et al. 2019).

We conducted an ecological survey across 385 sampling sites in Ningbo from May to June 2024 (Figure 1). All sites were in lotic river habitats (excluding reservoirs) and spanned a wide range of land‐use types, including forested, agricultural, and urban sites, representing a clear gradient of anthropogenic disturbance.

**FIGURE 1:**
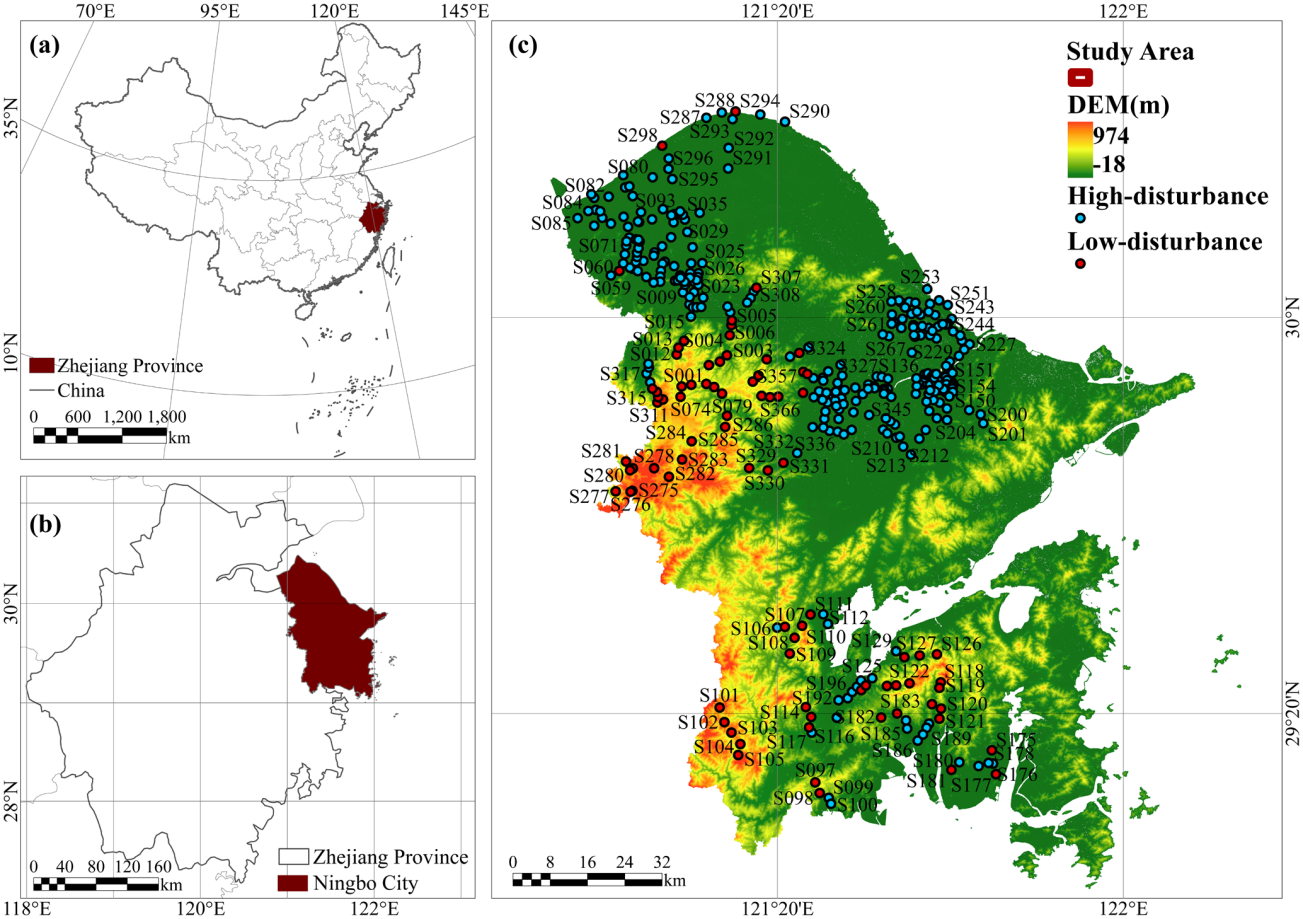
Geographical location and topographical features of the study area. (a) Location within China; (b) Location within Ningbo; (c) Sampling points are superimposed on the Digital Elevation Model (DEM) of Ningbo. Blue dots represent sampling points with high‐disturbance, while red dots indicate sampling points with lowdisturbance.

### 2.2 Sampling and Identification of Macroinvertebrates

Macroinvertebrate sampling was conducted from May to June 2024. Because habitats varied at each site, Surber nets were used at small‐stream sampling sites, while hand‐held nets were used at large‐river sampling sites to collect macroinvertebrates. We collected samples in triplicate at each site and combined them into one composite sample to ensure adequate representativeness. We sieved sediment samples through a 40‐mesh screen and washed them at the sampling site. All specimens were separated and sorted using white ceramic trays, then fixed and stored in a 75% ethanol solution (Tang et al. 2025). We removed surface fixatives from the collected macroinvertebrates using filter paper. Species classification and identification of macroinvertebrates were conducted in the laboratory using a dissecting microscope (XTL‐165 series microscope, Jiangxi Phoenix Optical Technology Corporation, China). When specimens were intact, macroinvertebrates were identified to the lowest feasible taxonomic level (typically species) (Dudgeon 1999; Epler 2001; Payakka and Prommi 2014; Merritt et al. 2017).

### 2.3 Environmental Factors

At each sampling site along the Ningbo River, we measured physicochemical variables using standardized instruments and procedures. Water quality parameters, including conductivity (Cond), dissolved oxygen (DO), water temperature (WT), and pH, were determined in situ using a YSI Professional Plus multiparameter meter (Yellow Springs, Ohio, USA). Flow velocity was measured near the sampling locations with a Global Water Flow Probe FP201 (Global Water Instrumentation, Gold River, California, USA). In addition, water samples were collected in 100 mL bottles at each site and stored at 0°C–4°C prior to laboratory analysis. The concentrations of non‐purgeable organic carbon (NPOC), concentrations of total phosphorus (TP), total nitrogen (TN), ammonia nitrogen (NH_3_‐N), nitrate nitrogen (NO_3_‐N), and chemical oxygen demand (COD_Mn_) were analyzed following the “Surface Water Environmental Quality Standards of the People's Republic of China” (GB3838‐2002). The nitrogen‐to‐phosphorus ratio (NPR) was calculated as TN divided by TP.

### 2.4 Species Traits

Examining the relationships between macroinvertebrate traits and environmental adaptation provides insight into the intensity of anthropogenic disturbance in river ecosystems. To assess variations in the trophic structure of macroinvertebrate communities, organisms were grouped into five functional feeding groups (FFGs) according to Heino (2005): shredders, scrapers, collector‐gatherers (gatherers), collector‐filterers (filterers), and predators. These functional groups reflect differences in food resource use and feeding strategies among macroinvertebrates (Cummins et al. 2005). Shredders consume coarse‐grained organic matter and transform it into fine‐grained organic matter. Filterers prey on suspended fine‐grained organic matter, while gatherers feed on organic detritus. Scrapers consume small algae, fungi, and bacteria attached to the substrate. Predators feed on small organisms. Table S2 details the FFG classification for macroinvertebrates used in this study.

### 2.5 Data Analysis

Land‐use data were obtained from the GlobalLand30 dataset, which provides global land‐cover information at a spatial resolution of 30 m and was developed using multi‐source remote‐sensing imagery (Wang et al. 2022). The data were processed through preprocessing, classification, post‐processing, and accuracy assessment, followed by hierarchical land‐use classification. Using ArcGIS, we established a 1‐km circular buffer around each sampling site and calculated the proportions of major land‐use categories, including forest, urban, and agricultural land, within each buffer. Field observations and satellite imagery were cross‐referenced to validate the land‐use classification. The percentages of artificial surfaces and agricultural land were subsequently summed to represent the proportion of anthropogenically influenced land use at each site. This composite index ranged from 0 to 100%. For the primary categorical analyses, sites with a combined proportion below 50% were classified as low‐disturbance sites (LD), whereas sites with a proportion of 50% or greater were classified as high‐disturbance sites (HD), following Xiao et al. (2025). The 50% threshold was used as an operational classification criterion to distinguish relatively low‐ and high‐disturbance sites, rather than as a universal ecological threshold. In addition, the continuous proportions of agricultural and urban land were retained for subsequent structural equation modeling (SEM), allowing their associations with macroinvertebrate diversity to be evaluated without categorical transformation.

We used abundance‐based macroinvertebrate data to quantify community structure using the Bray–Curtis dissimilarity matrix and tested significant differences among groups with Adonis (999 permutations) (Wu et al. 2022). Differences in macroinvertebrate community composition across sampling sites with varying anthropogenic disturbance levels were visualized using nonmetric multidimensional scaling (NMDS). The statistical significance of these differences was assessed via permutational multivariate analysis of variance (PERMANOVA) (Eskelinen and Harrison 2015). All analyses were performed using the *vegan* package in R (Oksanen et al. 2021).

To assess the impacts of different anthropogenic disturbances on macroinvertebrates, we examined alpha diversity and the proportions of different FFGs. Representative alpha diversity indices were calculated as follows: we used the diversity function in the R *vegan* package to compute taxonomic richness (TRic) and Pielou's evenness (TEve). Functional richness (FRic) and functional evenness (FEve) were calculated using the *dbFD* function from the R package *FD* (Laliberté and Legendre 2010). To assess the significance of alpha diversity differences among sites with varying anthropogenic disturbances, we employed the Wilcoxon test (Darilay and Naranjo 2011). Subsequently, the Tukey–Kramer test was applied to examine differences in FFGs between the two groups (Driscoll 1996).

SEM was used to evaluate the potential direct and indirect associations among anthropogenic land use, local environmental conditions, and macroinvertebrate taxonomic and functional diversity (Grace 2006). The conceptual model was specified a priori based on established ecological relationships among land use, local physicochemical conditions, and aquatic biodiversity. Specifically, agricultural and urban land use were hypothesized to be associated with macroinvertebrate diversity both directly and indirectly through changes in local environmental conditions. All variables included in the SEM were treated as observed variables. Agricultural and urban land‐use proportions were included as observed land‐use predictors, while environmental variables and the four diversity metrics (TRic, TEve, FRic, and FEve) were treated as observed variables. The initial pool of candidate environmental variables included water temperature, dissolved oxygen, conductivity, velocity, pH, COD_Mn_, TN, TP, NO_3_‐N, NH_3_‐N, NPR, and NPOC. We selected these variables based on their ecological relevance to river physicochemical conditions, nutrient availability, habitat quality, and organic‐matter conditions, as well as their potential relationships with macroinvertebrate communities.

Prior to SEM construction, candidate environmental variables were examined for multicollinearity. Variance inflation factors (VIFs) were used to identify highly collinear variables, and variables with substantial redundancy were not retained simultaneously in the final models (Lin et al. 2024). Based on this screening procedure, pH, NO_3_‐N, and NPOC were retained as environmental variables in the final SEMs. These variables represent complementary aspects of water chemistry and organic‐resource conditions that may mediate associations between anthropogenic land use and macroinvertebrate diversity. Piecewise SEM was conducted using the *psem* function in the *piecewiseSEM* package (Lefcheck 2016). Model fit was evaluated using Fisher's C statistic (*p* > 0.05) and the associated probability value. We also considered the Akaike information criterion (AIC) when evaluating model parsimony. We specified pathways according to the a priori conceptual framework rather than adding them solely on the basis of statistical significance. Standardized path coefficients were used to compare the strength and direction of associations among variables. Direct effects represented the standardized association between a predictor and its directly linked response variable, whereas indirect effects represented associations transmitted through one or more intermediate environmental variables (Gao et al. 2021). Total effects represented the combined direct and indirect associations.

Before SEM analysis, predictor variables were standardized to facilitate comparison among path coefficients. Diversity indices and selected environmental variables were log‐transformed where necessary to improve their distributional properties and satisfy the assumptions of linear modeling. All statistical analyses were conducted in R version 4.3.3 (R Core Team 2024).

## 3 Results

### 3.1 Environmental Parameters

Table S1 summarizes the environmental parameters of river reaches subjected to different levels of anthropogenic disturbance, including physicochemical water variables and land‐use characteristics (mean ± standard deviation). Pronounced differences in environmental conditions were observed between disturbance levels, with overall water quality showing a clear deterioration from low‐ to high‐disturbance zones (Figure 2).

**FIGURE 2:**
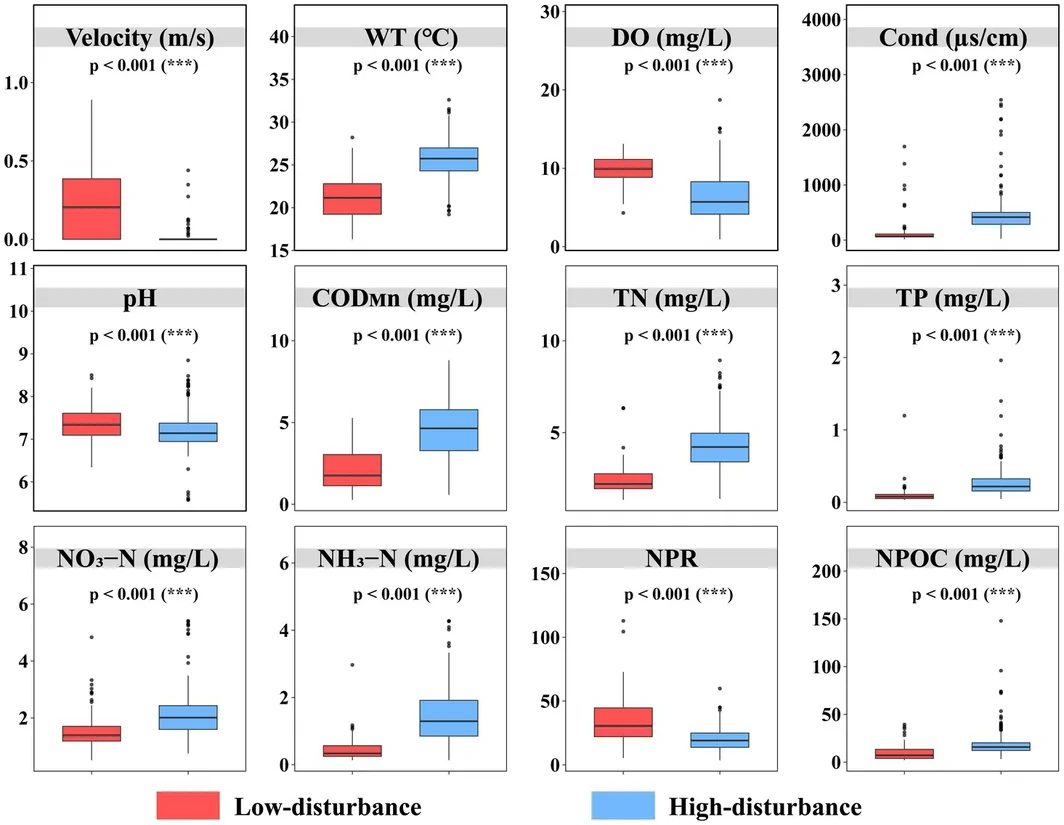
Results of the main abiotic factors for the low‐ and high‐disturbance sites, respectively. Boxes show interquartile ranges (25th and 75th percentiles) and medians (middle lines); whiskers show non‐outlier ranges beyond the boxes, and dots indicate outliers. Conductivity: Cond; Dissolved oxygen: DO; Water temperature: WT; Flow velocity: Velocity; Non‐purgeable organic carbon: NPOC; Total phosphorus: TP; Total nitrogen: TN; Ammonia nitrogen: NH_3_‐N; Nitrate nitrogen: NO_3_‐N; Chemical oxygen demand: COD_M_n; NPR: TN divided by TP. Asterisks indicate statistically significant differences between low‐ and high‐disturbance sites (*p < 0.05, **p < 0.01, and ***p < 0.001).

River reaches in high‐disturbance sites were characterized by greater channel dimensions and elevated nutrient and organic matter concentrations, with mean. Water temperature (25.71°C ± 2.32°C) and Cond (450.86 ± 341.83 μs/cm) were also relatively high. Nutrient enrichment was evident, as indicated by increased concentrations of NPOC (18.31 ± 12.68 mg/L), COD_Mn_ (4.55 ± 1.63 mg/L), NO_3_‐N (2.11 ± 0.79 mg/L), NH_3_‐N (1.43 ± 0.77 mg/L), TN (4.29 ± 1.25 mg/L), and TP (0.27 ± 0.19 mg/L).

In contrast, low‐disturbance reaches generally occurred at higher velocities (0.24 ± 0.23 m/s) and showed better water quality, with near‐neutral pH (7.34 ± 0.40), relatively high DO (9.72 ± 1.82 mg/L), and markedly higher NPR (34.38 ± 18.52). Additionally, forest cover peaks in low‐disturbance sites, while agricultural land and impervious surface sites are higher in high‐disturbance zones.

### 3.2 Community Composition of Macroinvertebrates Under Different Anthropogenic Disturbances

Our survey found that the macroinvertebrate community comprised organisms from 4 phyla, 8 classes, 24 orders, 91 families, and 211 species (Table S3). Common orders included Mesogastropoda, Decapoda, Diptera, Ephemeroptera, Tubificida, Odonata, and others. The species composition bar chart (Figure 3b) shows that low‐disturbance sites were primarily composed of Mesogastropoda, Ephemeroptera, Decapoda, Diptera, Odonata, and others. At high‐disturbance sampling sites, Mesogastropoda accounted for the largest proportion, followed by Decapoda, Diptera, Tubificida, and others. At high‐disturbance sites, the proportion of pollution‐sensitive taxa such as Ephemeroptera and Odonata decreased.

**FIGURE 3:**
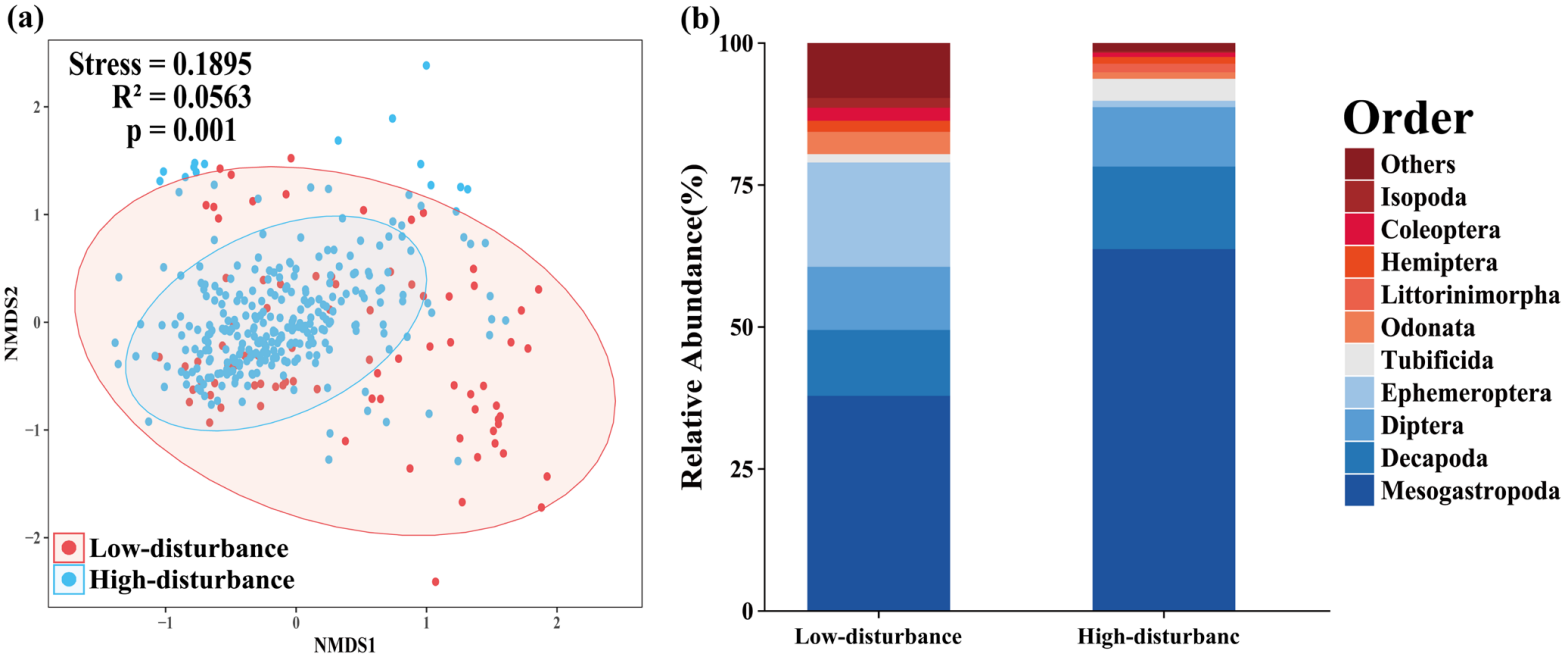
NMDS plots of macroinvertebrate communities (a) and their relative abundance chart (b) at sampling sites with low‐and high‐disturbance. Different colors in plot (a) represent sampling sites with varying disturbance levels: Red (low‐disturbance), blue (high‐disturbance). Stacked bar charts in plot (b) display the relative abundance of the top 10 macroinvertebrate taxa at these sampling sites, with less abundant taxa grouped under “Others”.

NMDS analysis yielded a stress value of 0.1895, which falls within the generally acceptable range (< 0.2) but is relatively close to the upper limit, indicating that the two‐dimensional ordination provides a reasonable, although not fully precise, representation of the multivariate community relationships (Figure 3a). Therefore, the spatial configuration of individual sampling points in the NMDS plot should be interpreted with some caution, particularly with respect to fine‐scale differences among sites. Nevertheless, the NMDS ordination showed a clear separation in community composition between low‐ and high‐disturbance sites. Permutational multivariate analysis of variance (PERMANOVA) further confirmed that macroinvertebrate community composition differed significantly between disturbance levels (*p* = 0.001, *R* ^2^ = 0.0563), with disturbance level explaining 5.63% of the variation in community composition. These results support our first hypothesis (H1) that macroinvertebrate community composition differs between low‐ and high‐disturbance sites.

### 3.3 Macroinvertebrate FFGs Across Different Disturbance Levels

Macroinvertebrate functional feeding groups (FFGs) differed significantly between low‐ and high‐disturbance sites (Figure 4). Filterers, Gatherers, Predators, Scrapers, and Shredders all showed significant differences between the two disturbance levels (*p* < 0.05). Filterers, Gatherers, and Predators were more abundant at low‐disturbance sites than at high‐disturbance sites. For Gatherers and Predators, the median relative abundances were 0.108 and 0.0769 at low‐disturbance sites, respectively, compared with 0 at high‐disturbance sites. For Filterers, the median relative abundance was 0 at both disturbance levels; however, the distribution differed significantly between the two groups, with more non‐zero observations and generally higher values at low‐disturbance sites. In contrast, Scrapers and Shredders were more abundant at high‐disturbance sites, with median relative abundances of 0.721 and 0.100, respectively, compared with 0.513 and 0.0449 at low‐disturbance sites (*p* < 0.05). These results indicate that disturbance levels were associated with a shift in the relative abundance distribution of FFGs, supporting the second hypothesis.

**FIGURE 4:**
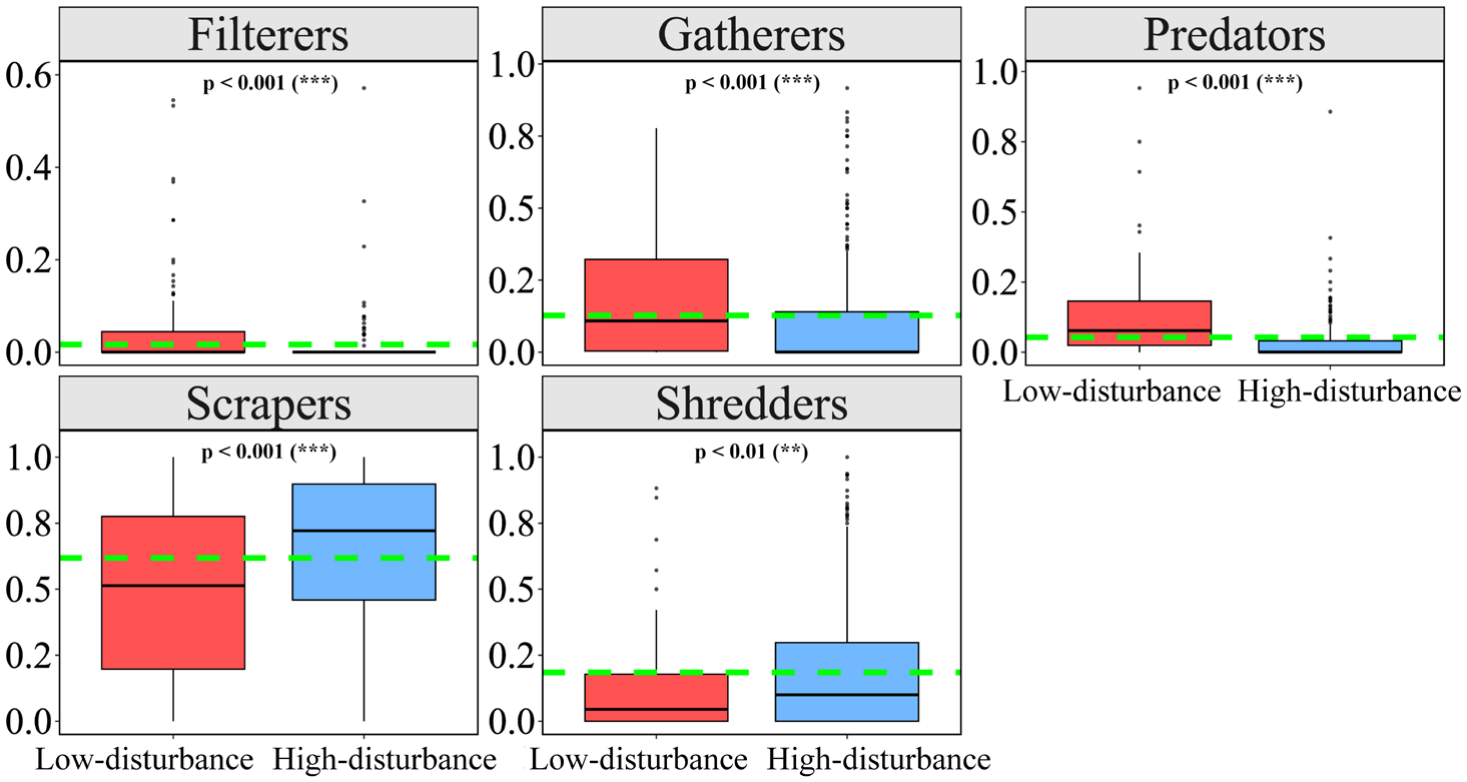
Box plots of relative abundance of functional feeding groups of macroinvertebrates at sampling sites with low‐ and high‐disturbance. Horizontal lines represent the median, while green dashed lines denote the mean. Outliers are indicated by dots. Significant statistical differences between sampling sites are indicated by *p*‐values, with ** and *** denoting *p* < 0.01 and *p* < 0.001, respectively.

### 3.4 Alpha Diversity of Macroinvertebrates Across Different Disturbance Levels

Overall, alpha diversity patterns suggest that high‐disturbance sites were characterized by a generally degraded functional condition, rather than improved community structure (Figure 5).

**FIGURE 5:**
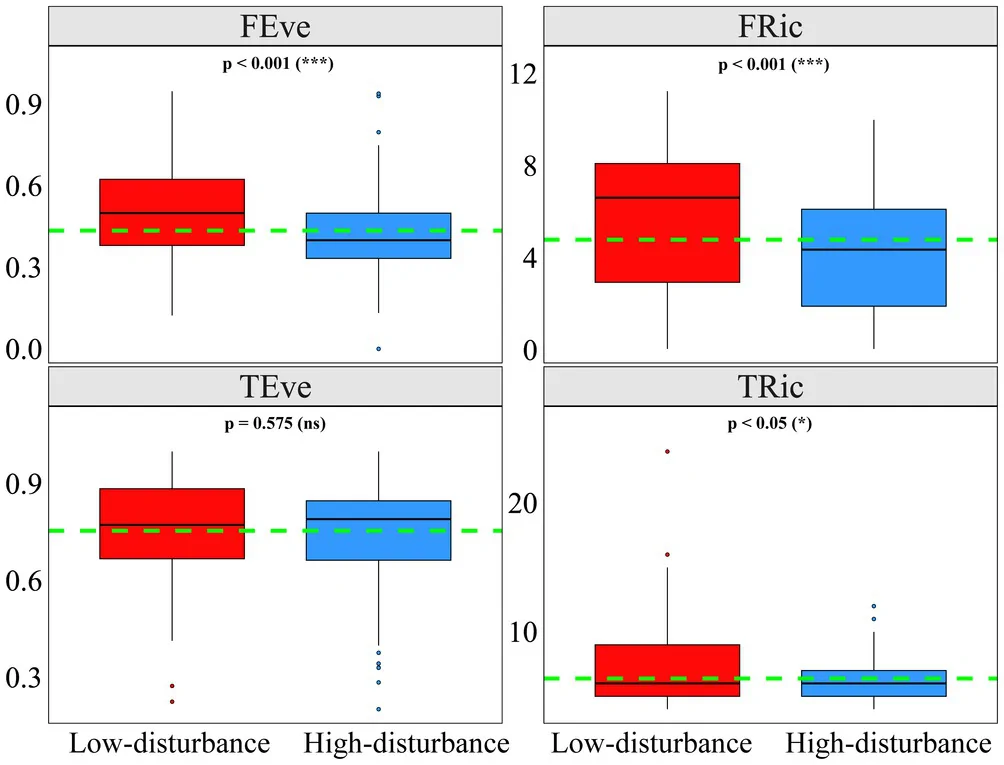
Box plots of alpha diversity indices between low‐ and high‐disturbance sampling sites. The Wilcoxon test was used to assess differences between low‐disturbance and high‐disturbance sampling sites; *p*‐values indicate statistical significance (*p* > 0.05 indicates no significant difference; *p* < 0.05 indicates significant difference), with *, **, and *** denoting *p* < 0.05, *p* < 0.01, and *p* < 0.001, respectively. Dots indicate outliers; the green dashed line represents the mean. FEve, functional evenness; FRic, functional richness; TEve, taxonomic evenness; TRic, taxonomic richness.

Functional diversity indices declined under high‐disturbance, with lower FEve (0.409 vs. 0.51) and lower FRic (4.108 vs. 6.473) than at low‐disturbance sites (*p* < 0.001), indicating reduced functional trait dispersion and a contraction of functional niche space in highly disturbed reaches.

TRic also differed significantly between disturbance levels (*p* < 0.05), although the median values were similar between low‐ and high‐disturbance sites. This result indicates that the significant difference in TRic was driven by differences in the overall distribution of taxonomic richness rather than by a substantial difference in the median value.

TEve showed a slightly higher median value at high‐disturbance sites (0.796 vs. 0.761), but the difference was not significant (*p* = 0.575), suggesting that increased disturbance did not enhance community balance but may instead reflect environmental filtering favoring tolerant taxa.

### 3.5 Relationships Among Land Use, Environmental Factors and Alpha Diversity

This study employed SEM to quantify the direct, indirect, and total effects of urban and agricultural land use types on diversity (FRic, FEve, TRic, TEve) through water quality indicators (pH, NO_3_‐N, NPOC). The results showed that both urban and agricultural land use exerted significant negative direct effects on FRic, FEve, and TRic, with urban land use having the strongest direct impact on TRic. In contrast, neither land use type had a significant direct effect on TEve. Across all models, indirect effects mediated by water quality were consistently weak, indicating a limited role in transmitting land‐use impacts to ecosystem function.

For richness (Figure 6a,c), the environmental factor NPOC exhibited a sustained and strong positive total effect on both FRic and TRic, acting as the most prominent positive driver of these traits, while the total effects of NO_3_‐N and pH were relatively weak. For evenness (Figure 6b,d), the total effects of NPOC and pH were consistently negative but of low magnitude, indicating limited negative impacts on functional evenness, and the effects of NO_3_‐N were negligible.

**FIGURE 6:**
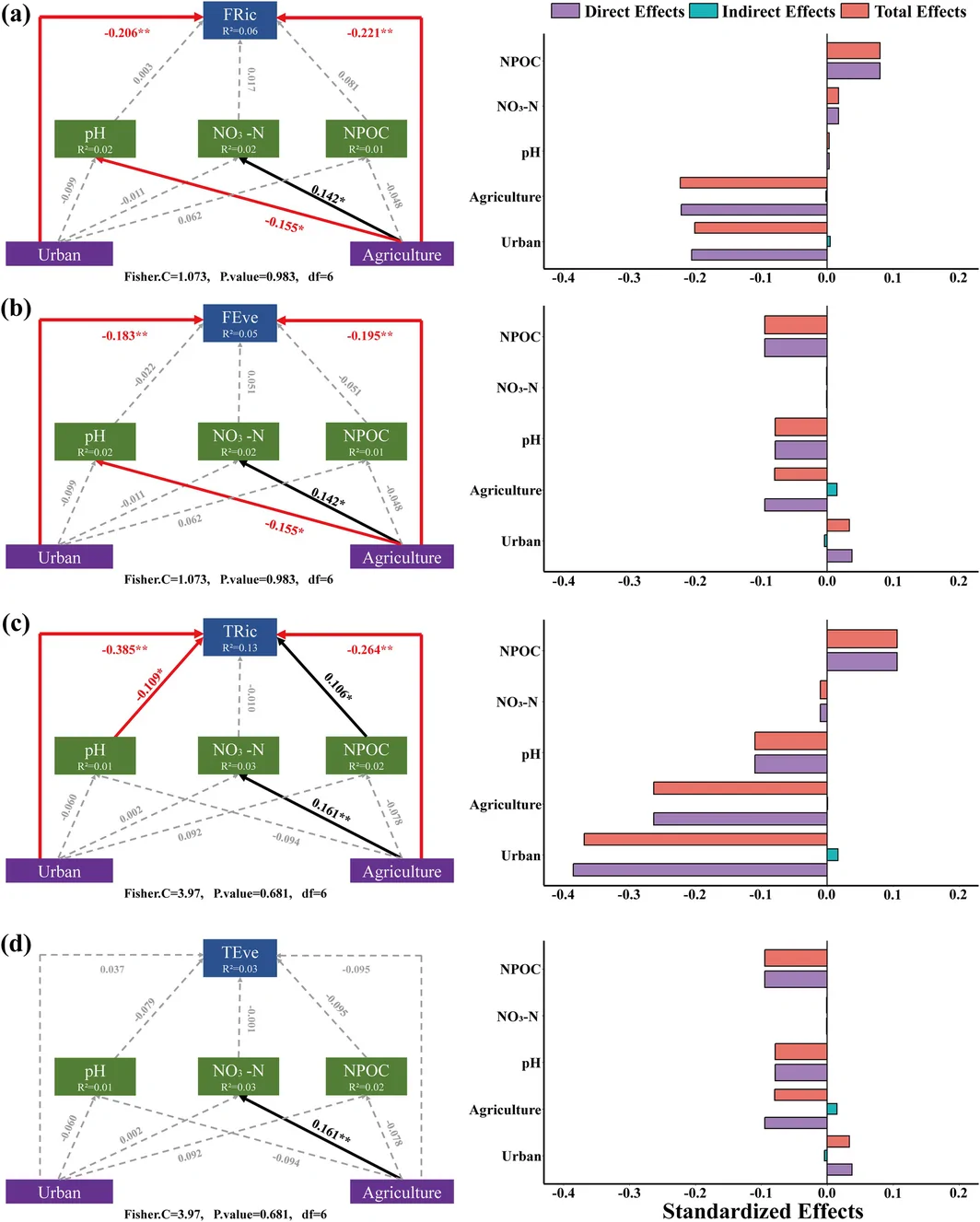
SEM illustrating direct or indirect effects of land use on (a) FRic, (b) FEve, (c) TRic, and (d) TEve. Purple indicates land use variables; green denotes local environmental variables. Black, red, and dashed arrows represent significant positive, negative, and non‐significant path relationships, respectively. The goodness‐of‐fit statistics for each model are displayed below the model. Asterisks indicate the significance of path coefficients: *p < 0.05, **p < 0.01. The right side shows corresponding SEM standard effect statistics: Direct effects (purple bar charts), indirect effects (blue bar charts), total effects (orange bar charts). *p* > 0.05 indicates model significance, and model fit indices suggest good model fit.

Model fit statistics (*R* ^2^) further revealed that TRic was the most responsive trait to land use changes (*R* ^2^ = 0.13), while TEve was the least sensitive (*R* ^2^ = 0.03). Collectively, these findings confirm that urban and agricultural land use are the primary drivers of reduced aquatic ecosystem function, with direct negative impacts far outweighing the limited indirect effects mediated by water quality. This is consistent with some of the assumptions in H4.

## 4 Discussion

### 4.1 Anthropogenic Disturbance Affects Macroinvertebrate Community Composition

Our research shows that habitat alterations under varying degrees of anthropogenic disturbance significantly reshape macroinvertebrate community composition. In this study, macroinvertebrate communities primarily comprised several major orders, notably Mesogastropoda, Decapoda, Diptera, and Ephemeroptera. Comparative analysis between sampling sites with varying disturbance levels showed that Mesogastropoda constituted a significantly higher proportion at high‐disturbance sites than at low‐disturbance sites. This may be attributed to their robust ecological adaptability and ability to thrive in diverse habitats, enabling rapid survival and reproduction across different regions (Hermosillo‐Núñez et al. 2023). Conversely, the proportion of Ephemeroptera at low‐disturbance sampling sites was significantly higher than at high‐disturbance sites. This indicates that the distribution of pollution‐sensitive Ephemeroptera taxa is primarily related to anthropogenic land‐use intensity (Ferreira et al. 2014). These taxa are typical water‐quality indicator species, showing high sensitivity to factors such as dissolved oxygen levels, pollutant concentrations, and flow velocity in water bodies. In this study, the low‐disturbance group, where Ephemeroptera occurred, exhibited significantly higher flow velocity and dissolved oxygen levels than the high‐disturbance group (Figure 2), along with significantly greater forest cover (Figure S1). This aligns with findings from previous studies (Pond 2010; Arimoro and Muller 2010; Zuedzang Abessolo et al. 2021).

Forest sites typically provide stable and heterogeneous habitats characterized by rich riparian vegetation, low sediment input, and better water quality, favoring diverse communities dominated by Ephemeroptera taxa (Dos Reis Oliveira et al. 2020). Agricultural and urbanized sites introduce greater environmental disturbances such as sedimentation, nutrient enrichment, and chemical pollution, which degrade habitat quality and lead to the dominance of pollution‐tolerant taxa (Gerth et al. 2017; Wang et al. 2025). Consequently, this can influence species composition between two distinct anthropogenic disturbance groups.

These patterns broadly align with responses reported for both urban and agricultural streams, where intensified land use can impose multiple interacting pressures on stream communities through altered hydrology, sediment dynamics, nutrient enrichment, and habitat degradation (Sabat‐Bonilla et al. 2026). In particular, the observed replacement of disturbance‐sensitive taxa by more tolerant taxa is consistent with the broader “urban stream syndrome” framework (Brunt et al. 2025), which describes the cumulative effects of urbanization on stream hydrology, water quality, channel habitat, and biological communities. Although our study did not directly quantify all of these stressors, the consistent differences in community composition between low‐ and high‐disturbance sites suggest that intensified agricultural and urban land use may act as an environmental filter on macroinvertebrate assemblages. Similar community responses reported in agricultural streams further indicate that different forms of land‐use intensification can generate cumulative pressures that simplify stream communities.

### 4.2 Macroinvertebrate FFGs Affected by Different Anthropogenic Disturbances

Macroinvertebrate FFGs are highly sensitive to disturbances within communities, and their trophic ecology provides an effective approach for analyzing dynamic changes in community structure within fragile ecosystems under human disturbance (Heino 2005; Yaagoubi et al. 2023). Differences in FFGs among macroinvertebrates across various anthropogenic disturbance types may relate to specific food resource availability and human activity intensity in different regions of Ningbo. This study found that in low‐disturbance sites of Ningbo rivers, filterers, gatherers, and predators accounted for a higher proportion, while scrapers and shredders dominated in high‐disturbance zones.

Low‐disturbance sites typically have more stable hydrological conditions and lower suspended sediment concentrations, which support filterers (such as Ephemeroptera and Bivalves) in their feeding structures. These findings align with Vaughn (2018), confirming that filterers are highly sensitive to water turbidity and suspended particles. High turbidity clogs their filtering organs, reducing feeding efficiency. Furthermore, low‐disturbance sites typically receive continuous litterfall input, providing filterers with a stable source of fine organic particles (Wallace and Webster 1996). Gatherers (e.g., Chironomids, Oligochaetes) in low‐disturbance sites can access fine organic particles abundant in riverbed sediments, providing them with rich food resources. These findings support Richardson and Mackay's (1991) view that stable hydrological conditions and continuous organic inputs are key factors in maintaining the diversity and abundance of gatherers. Highly disturbed sites, where agricultural activities and urban sewage discharge alter sediment organic matter composition, may be unfavorable for collector survival. Complex habitat structures in low‐disturbance sites (e.g., rock piles, deadwood accumulations) provide excellent hiding places and diverse prey resources, benefiting predators such as dragonfly larvae and beetles. This finding aligns with Smith et al. (2003), who identified habitat complexity as a primary determinant of predator distribution. In highly disturbed sites, pollutant accumulation and habitat simplification reduce prey resources, leading to a corresponding decrease in predator abundance.

Highly disturbed sites are often associated with agricultural fertilization and domestic sewage inputs, which elevate nitrogen and phosphorus levels in water bodies. This promotes algal and epiphytic growth, providing a rich food base for scrapers (such as Mesogastropoda). These scrapers are more tolerant and adaptable to poorer environmental conditions (Lenat and Crawford 1994), resulting in higher scraper densities compared to other FFGs. These findings align with Wellnitz and Poff (2006), who documented a significant increase in the proportion of scrapers in eutrophic waters, particularly in watersheds heavily impacted by agriculture. Furthermore, the study revealed that crop debris left after harvest and large‐particle organic matter from domestic sewage inputs provide alternative resources for shredders (e.g., stoneflies, certain Trichoptera larvae). This result aligns with Dobson (1994), who indicated that in disturbed rivers, introduced organic debris can support the maintenance or even increase of shredder communities.

The observed changes in FFG structure may also affect the resistance and resilience of stream communities under continued environmental stress. The greater representation of tolerant feeding groups under high‐disturbance may allow some taxa to persist under altered environmental conditions, but such persistence should not necessarily be interpreted as evidence of greater ecological resilience (Miller et al. 2018). Rather, the reduction or loss of sensitive taxa and changes in functional‐group composition may reduce the range of ecological strategies available to the community when facing subsequent disturbances (Li et al. 2021). Thus, functional reorganization under intensified land use may influence stream communities' capacity to maintain ecological functions or recover following disturbance (Thornhill et al. 2018). However, because we did not directly measure resistance and resilience in our spatial study, treat these interpretations as potential ecological implications rather than direct evidence of altered resistance or recovery capacity.

### 4.3 Impact of Anthropogenic Disturbance on Macroinvertebrate Alpha Diversity

Functional alpha diversity assesses functional strategy diversity within a community based on species' functional traits, focusing on the spatial distribution of ecological functions and resource‐utilization efficiency. Taxonomic alpha diversity denotes species composition diversity within a community, measured by species richness, the Shannon‐Wiener index, and species evenness (Pitacco et al. 2019; Zhou et al. 2022; Tsang et al. 2025). Land use types shape macroinvertebrate community alpha diversity through land‐use intensity, resource availability, and environmental filtering (Wang et al. 2025). In this study, macroinvertebrate alpha diversity under different anthropogenic disturbance types in the Ningbo region reflects distinct screening mechanisms under varying anthropogenic activity pressures.

In terms of functional diversity, the FRic index reflects the extent to which organisms occupy functional niches within a community, indicating the degree of ecological space utilization. FRic index values decrease from less disturbed to more disturbed river segments. This suggests that ecological space utilization is higher in less disturbed segments and lower in highly disturbed segments. Furthermore, the FRic index has been proposed as a key characteristic of biological communities that can predict ecosystem reliability (Mason et al. 2005; Wu et al. 2024). The lower FRic index observed in highly disturbed sites indicates ecological niche vacancies, making these regions susceptible to invasive species colonization. The FEve index measures the uniformity of species functional traits within ecological space distribution. Low functional evenness indicates that certain ecological niches are occupied but underutilized. This typically reduces ecosystem productivity and stability while increasing the likelihood of alien species colonization (Mason et al. 2005). The high FEve levels observed in low‐disturbance sites suggest more comprehensive and efficient use of land resources, whereas high‐disturbance sites show insufficient resource utilization.

In terms of taxonomic diversity, low‐disturbance sites show higher taxonomic richness. This is attributed to the relatively intact vegetation cover and hydrological conditions typically found in low‐disturbance zones, which reduce soil erosion and pollutant inputs while providing stable habitats for macroinvertebrates (Lin et al. 2022; Paiva et al. 2025). In highly disturbed sites, organic matter inputs to water bodies, habitat structure, and species interactions change. Many species may be unable to survive under extreme conditions, leading to a significant decline in species richness. This aligns with Liu et al. (2022).

However, our study found no significant difference in TEve between the low‐disturbance and high‐disturbance groups (Figure 4). Typically, disturbance affects species distribution and dominance, thereby altering taxonomic evenness (Liu et al. 2022). Yet in this study area, land‐use intensity may have been insufficient to cause significant shifts in dominant species. This phenomenon may also reflect the adaptability of macroinvertebrate communities. Some macroinvertebrate species, particularly those with stronger adaptive capacity, can maintain relatively balanced taxonomic structures under varying disturbance intensities, thereby sustaining high taxonomic evenness. As noted by Villéger et al. (2008), functional evenness (FEve) reflects the continuity and regularity of resource utilization within a community. When environmental filtering is intense, species possessing certain maladaptive traits disappear (Ephemeroptera), regardless of whether they were originally common or rare. This creates gaps in functional space, reducing FEve, but does not necessarily mean the remaining species become uneven in abundance.

These contrasting responses also have implications for biomonitoring in agricultural and urban streams. Our results suggest that taxonomic richness or evenness alone may not fully capture the ecological effects of intensified land use, because functional diversity and FFG composition can reveal additional dimensions of community reorganization. Therefore, combining taxonomic composition, functional diversity, and FFG structure within a multi‐metric monitoring framework may provide a more comprehensive assessment of ecological degradation and recovery (Zhang et al. 2024). Such an approach may be particularly useful for detecting functional simplification when changes in taxonomic richness are relatively limited.

### 4.4 Land‐Use Intensity Directly and Indirectly Influences Alpha Diversity

Our results support H4 by showing that anthropogenic land use was associated with macroinvertebrate alpha diversity through both direct and indirect pathways, although the strength of these pathways varied among biodiversity dimensions and land‐use types. Rather than indicating a single pollution‐driven mechanism, the SEM results suggest that land‐use effects may operate through a combination of habitat alteration and changes in local resource conditions. This pattern is consistent with an environmental‐filtering framework, in which landscape modification alters the local environmental template and resource environment, thereby influencing which taxa and functional strategies can persist. Because our study used observational data, we interpret the SEM paths as statistical associations and potential pathways rather than definitive causal effects.

The continuous land‐use analysis complemented the categorical comparison between LD and HD sites. Agricultural and urban land‐use proportions were negatively associated with both taxonomic and functional alpha diversity, with direct paths accounting for a larger proportion of the observed associations than indirect paths. This pattern suggests that the measured physicochemical variables alone cannot fully explain the relationship between anthropogenic land use and biodiversity (Heuss et al. 2019). Direct associations may reflect unmeasured or incompletely captured changes in habitat structure, channel morphology, riparian condition, and hydrological connectivity that commonly accompany agricultural and urban development (Uno et al. 2025; Wei et al. 2025). Previous studies have similarly emphasized that land‐use change can affect macroinvertebrate communities through habitat simplification and channel modification in addition to changes in water quality (Gücker et al. 2024).

The stronger negative association between urban land use and taxonomic alpha diversity further suggests that different land‐use types may impose different environmental constraints on macroinvertebrate communities. Urban development often brings increased impervious surfaces, bank modification, and altered runoff and hydrological connectivity, which can change benthic habitat conditions and reduce the availability of suitable habitats for sensitive taxa (Luo et al. 2018). In our study, the stronger association of urban land use with taxonomic diversity may therefore reflect the cumulative influence of multiple forms of urban habitat modification (Figure 6c). However, because we did not directly quantify these habitat characteristics in the present study, this interpretation should be considered a potential mechanism rather than a demonstrated causal pathway.

Among the measured environmental variables, NPOC showed a significant positive association with richness‐related alpha diversity and formed an important indirect pathway linking agricultural land use to macroinvertebrate diversity (Figure 6a,c). This result highlights the potential role of organic carbon availability as a resource‐related component of environmental filtering. Organic carbon provides an important energy source for lotic food webs and can support detritivores and collector taxa while contributing indirectly to energy transfer to higher trophic levels (Bundschuh and McKie 2016; Demars et al. 2021). Agricultural land use may alter riparian vegetation and organic‐matter inputs, potentially changing both the quantity and quality of organic resources available to stream communities. The positive NPOC–diversity association observed here therefore provides a plausible ecological pathway through which agricultural land use may indirectly affect macroinvertebrate diversity (Bohn et al. 2024).

In contrast, NO_3_‐N did not show significant direct associations with the diversity metrics in the SEM. This result indicates that nitrate concentration alone did not explain substantial variation in alpha diversity within the environmental range observed across our study sites. It also suggests that nutrient concentrations alone may not fully capture the ecological response to anthropogenic land use. Habitat conditions and resource availability may interact with water chemistry to shape macroinvertebrate communities, particularly when nutrient concentrations remain within a range that does not impose strong physiological or ecological constraints. Similar findings have been reported along low‐to‐moderate nutrient gradients, where habitat structure and energy availability can contribute substantially to variation in macroinvertebrate diversity (Gál et al. 2019; Chen et al. 2025).

Overall, these findings provide a more integrated interpretation of H4. Anthropogenic land use was associated with reduced macroinvertebrate alpha diversity primarily through direct pathways, while environmental mediation, particularly through NPOC, contributed to a smaller but ecologically meaningful component of the observed relationships (Cui et al. 2026). This pattern supports a multiple‐pathway view of land‐use impacts: landscape development may alter biodiversity not only through measurable changes in water chemistry but also through habitat modification and changes in resource availability (Kramer et al. 2026). Therefore, assessments of riverine biodiversity under land‐use change should consider both the physical habitat template and the resource environment rather than relying exclusively on conventional water‐quality indicators (Moi et al. 2024). From a management perspective, maintaining riparian vegetation, organic‐matter inputs, and habitat complexity from a watershed view may help sustain macroinvertebrate biodiversity in human‐dominated river networks (Cai 2026; Fanteso et al. 2026; Xu 2026).

## 5 Conclusion

This study assessed the effects of anthropogenic disturbance on macroinvertebrate community structure and diversity in Ningbo River ecosystems under different land‐use conditions. We recorded 211 macroinvertebrate species. Low‐disturbance sites supported higher proportions of pollution‐sensitive taxa, whereas highly disturbed sites were dominated by disturbance‐tolerant groups, indicating clear shifts in community composition. Macroinvertebrate communities exhibited distinct spatial differentiation across the land‐use intensity gradient, with significantly higher taxonomic and functional alpha diversity in low‐disturbance sites. Functional structure also differed markedly, reflecting changes in energy pathways and habitat conditions as human disturbance increased. SEM further confirms that land use type affects macroinvertebrate diversity through both direct and indirect effects: it directly alters the riverine environment and indirectly modifies key environmental factors, with the direct effect more pronounced.

Overall, anthropogenic disturbance reshapes macroinvertebrate community composition, diversity, and functional organization through habitat alteration, highlighting its critical role in river ecosystem degradation. These findings provide scientific support for ecological assessment and river conservation management in Ningbo. Future studies should incorporate multi‐seasonal, long‐term, and multi‐taxa monitoring to better capture ecosystem responses to land‐use‐driven disturbances.

## Author Contributions

**Huawei Bao:** conceptualization (equal), data curation (equal), formal analysis (equal), investigation (equal), methodology (equal), visualization (equal), writing – original draft (equal). **Zongwei Lin:** investigation (equal), writing – review and editing (equal). **Zhijun Xia:** writing – review and editing (equal). **Min Zhang:** writing – review and editing (equal). **Xiao Du:** writing – review and editing (equal). **Collins Oduro:** investigation (equal), writing – review and editing (equal). **Sangar Khan:** investigation (equal), writing – review and editing (equal). **Naicheng Wu:** funding acquisition (lead), investigation (lead), methodology (lead), project administration (lead), supervision (lead), writing – review and editing (equal).

## Funding

This work was supported by National Natural Science Foundation of China (Nos. 52279068, 42350410434).

## Conflicts of Interest

The authors declare no conflicts of interest.

## Supporting information


**Table S1:** Abiotic factors (Mean ± SD) used in this study.
**Table S2:** Macroinvertebrates' functional trait groups, categories, codes, and descriptions used in this study.
**Table S3:** Macroinvertebrate species identified in this study. Details about functional feeding groups (FFGs) are as in Table S2.
**Figure S1:** Results of agriculture, impervious, and forest for the low‐disturbance and high‐disturbance sites, respectively. Boxes show interquartile ranges (25th and 75th percentiles) and medians (middle lines); whiskers are non‐outlier ranges beyond boxes, and dots are outliers.

## Data Availability

The data that support the findings of this study are available in the Supporting Information S1.
