# Supplementary material for: Differential Impacts of Anthropogenic Disturbance on Community Composition and Alpha Diversity of Macroinvertebrates at a Large Urban River System
Source: Ecol Evol. 2026 Oct 22;16(10):e74494. doi: 10.1002/ece3.74494 (PMC13688088; doi:10.1002/ece3.74494)
Supplement: Supplementary file 1 [file ECE3-16-e74494-s001.docx]

SUPPORTING INFORMATION

# Differential impacts of anthropogenic disturbance on community composition and alpha diversity of macroinvertebrates at a large urban river system

Huawei Bao^1,2^, Zongwei Lin^3^, Zhijun Xia^1^, Min Zhang^4^, Xiao Du^5^, Collins Oduro^1,2^, Sangar Khan^1,2^, Naicheng Wu^1,2*^

^1^ School of Geography and Remote Sensing, School of Civil Engineering and Future Cities, Ningbo University, Ningbo, China

^2^ Zhejiang-Germany Joint Laboratory on Remote Sensing of Coastal Ecosystem, Ningbo University, Ningbo, China

^3^ Department of Geosciences and Geography, University of Helsinki, Helsinki, Finland

^4^ State Key Laboratory of Water Cycle and Water Security, China Institute of Water Resources and Hydropower Research, Beijing, China

^5^ State Key Laboratory of Freshwater Ecology and Biotechnology - Ningbo Laboratory, Ningbo, China

**Corresponding author: Naicheng Wu (naichengwu88@gmail.com,* [*wunaicheng@nbu.edu.cn*](mailto:wunaicheng@nbu.edu.cn)*)*

**Table S1** Abiotic factors (Mean ± SD) used in this study.

| **Variables** | **Units** | **Abbreviation** | **All sites (n=385)** | **Low-disturbance (n=82)** | **High-disturbance (n=303)** |
| --- | --- | --- | --- | --- | --- |
| Forest | % | Forest | 19.35 ± 32.68 | 77.47 ± 21.44 | 3.62 ± 8.46 |
| Agriculture | % | Agriculture | 35.48 ± 29.58 | 14.24 ± 12.19 | 41.23 ± 30.28 |
| Impervious | % | Impervious | 41.34 ± 34.60 | 4.17 ± 7.47 | 51.41 ± 32.10 |
| Flow Velocity | m/s | Velocity | 0.06 ± 0.15 | 0.24 ± 0.23 | 0.01 ± 0.04 |
| pH | - | pH | 7.22 ± 0.43 | 7.34 ± 0.40 | 7.18 ± 0.44 |
| Water temperature | ◦C | WT | 24.76 ± 2.99 | 21.26 ± 2.59 | 25.71 ± 2.32 |
| Conductivity | µs/cm | Cond | 397.01 ± 350.89 | 151.03 ± 274.90 | 450.86 ± 341.83 |
| Dissolved oxygen | mg/L | DO | 7.02 ± 3.08 | 9.72 ± 1.82 | 6.29 ± 2.95 |
| Non-Purgeable Organic Carbon | mg/L | NPOC | 16.55 ± 12.35 | 10.06 ± 8.28 | 18.31 ± 12.68 |
| Chemical oxygen demand | mg/L | COD_Mn_ | 4.04 ± 1.84 | 2.14 ± 1.25 | 4.55 ± 1.63 |
| Amonia-nitrogen | mg/L | NH_3_-N | 1.22 ± 0.81 | 0.46 ± 0.37 | 1.43 ± 0.77 |
| Nitrate-nitrogen | mg/L | NO_3_-N | 1.99 ± 0.80 | 1.56 ± 0.68 | 2.11 ± 0.79 |
| Total nitrogen | mg/L | TN | 3.91 ± 1.39 | 2.49 ± 0.87 | 4.29 ± 1.25 |
| Total phosphorus | mg/L | TP | 0.23 ± 0.19 | 0.10 ± 0.13 | 0.27 ± 0.19 |
| TN/TP ratio | - | NPR | 22.97 ± 12.67 | 34.38 ± 18.52 | 19.88 ± 8.14 |

**Table S2** Macroinvertebrates’ functional trait groups, categories, codes, and descriptions used in this study.

| Functional feeding groups | **Categories** | **Codes** | **Descriptions** |
| --- | --- | --- | --- |
|  | Filters | FFG_fc | The FFG classification is based on the food consumed and also considers the morphological and behavioral characteristics used in the food acquisition. |
|  | Gathers | FFG_gc |  |
|  | Shredders | FFG_sh |  |
|  | Scrapers | FFG_sc |  |
|  | Predators | FFG_pr |  |

**Table S3** Macroinvertebrate species identified in this study. Details about functional feeding groups (FFGs), are as in Table S2.

| **Species code** | **Phylum** | **Class** | **Order** | **Family** | **Species** | **FFG** |
| --- | --- | --- | --- | --- | --- | --- |
| SM001 | Annelida | Clitellata | Rhynchobdellida | Glossiphoniidae | *Actinobdella sp.* | pr |
| SM002 | Annelida | Clitellata | Tubificida | Naididae | *Branchiura sowerbyi Beddard* | gc |
| SM003 | Annelida | Clitellata | Arhynchobdellida | Erpobdellidae | *Erpobdella sp.* | pr |
| SM004 | Annelida | Clitellata | Rhynchobdellida | Glossiphoniidae | *Glossiphonia nebulosa Kalbe* | pr |
| SM005 | Annelida | Clitellata | Rhynchobdellida | Glossiphoniidae | *Helobdella sp.* | pr |
| SM006 | Annelida | Clitellata | Tubificida | Naididae | *Limnodrilus hoffmeisteri* | gc |
| SM007 | Annelida | Clitellata | Tubificida | Enchytraeidae | *Marionina sp.* | gc |
| SM008 | Annelida | Clitellata | Tubificida | Naididae | *Monopylephorus limosus* | gc |
| SM009 | Annelida | Clitellata | Rhynchobdellida | Glossiphoniidae | *Placobdella sp.* | pr |
| SM010 | Annelida | Clitellata | Tubificida | Naididae | *Tubifex sp.* | gc |
| SM011 | Arthropoda | Insecta | Plecoptera | Perlidae | *Acroneuria sp.* | pr |
| SM012 | Arthropoda | Insecta | Plecoptera | Perlidae | *Agnetina sp.* | pr |
| SM013 | Arthropoda | Insecta | Plecoptera | Capniidae | *Allocapnia sp.* | sh |
| SM014 | Arthropoda | Insecta | Plecoptera | Chloroperlidae | *Alloperla sp.1* | gc |
| SM015 | Arthropoda | Insecta | Plecoptera | Chloroperlidae | *Alloperla sp.2* | gc |
| SM016 | Arthropoda | Insecta | Hemiptera | Naucoridae | *Ambrysus lunatus* | pr |
| SM017 | Arthropoda | Insecta | Ephemeroptera | Ameletidae | *Ameletus sp.1* | gc |
| SM018 | Arthropoda | Insecta | Ephemeroptera | Ameletidae | *Ameletus sp.2* | gc |
| SM019 | Arthropoda | Insecta | Coleptera | Ptilodactylidae | *Anchytarsus sp.* | sh |
| SM020 | Arthropoda | Insecta | Ephemeroptera | Potamanthidae | *Anthopotamus sp.* | gc |
| SM021 | Arthropoda | Insecta | Diptera | Limoniidae | *Antocha sp.* | gc |
| SM022 | Arthropoda | Insecta | [Trichoptera](https://en.wikipedia.org/wiki/Caddisfly) | Apataniidae | *Apatania sp.* | sc |
| SM023 | Arthropoda | Insecta | Trichoptera | Arctopsychidae | *Arctopsyche sp.* | fc |
| SM024 | Arthropoda | Insecta | Odonata | Coenagrionidae | *Argia sp.* | pr |
| SM025 | Arthropoda | Arachnida | Araneae | Dictynidae | *Argyroneta aquatica* | pr |
| SM026 | Arthropoda | Insecta | Diptera | Athericidae | *Atherix sp.* | pr |
| SM027 | Arthropoda | Insecta | Ephemeroptera | Ephemerellidae | *Attenella sp.* | gc |
| SM028 | Arthropoda | Insecta | Ephemeroptera | Baetidae | *Baetis sp.* | gc |
| SM029 | Arthropoda | Insecta | Hemiptera | Belostomatidae | *Belostoma Latreille* | pr |
| SM030 | Arthropoda | Insecta | Hemiptera | Belostomatidae | *Belostoma sp.* | pr |
| SM031 | Arthropoda | Insecta | Coleoptera | Hydrophilidae | *Berosus sp.* | gc |
| SM032 | Arthropoda | Insecta | Diptera | Ceratopogonidae | *Bezzia sp.* | pr |
| SM033 | Arthropoda | Insecta | Odonata | Aeshnidae | *Boyeria sp.* | pr |
| SM034 | Arthropoda | Insecta | Ephemeroptera | Caenidae | *Caenis sp.* | gc |
| SM035 | Arthropoda | Insecta | Odonata | Calopterygidae | *Calopteryx sp.* | pr |
| SM036 | Arthropoda | Insecta | Trichoptera | Leptoceridae | *Ceraclea sp.* | gc |
| SM037 | Arthropoda | Insecta | Diptera | Ulidiidae | *Chaetopsis apicalis* | gc |
| SM038 | Arthropoda | Insecta | Trichoptera | Hydropsychidae | *Cheumatopsyche sp.1* | fc |
| SM039 | Arthropoda | Insecta | Trichoptera | Hydropsychidae | *Cheumatopsyche sp.2* | fc |
| SM040 | Arthropoda | Insecta | Trichoptera | Philopotamidae | *Chimarra sp.1* | fc |
| SM041 | Arthropoda | Insecta | Trichoptera | Philopotamidae | *Chimarra sp.2* | fc |
| SM042 | Arthropoda | Insecta | Diptera | Chironomidae | *Chironomus sp.* | gc |
| SM043 | Arthropoda | Insecta | Plecoptera | Perlodidae | *Clioperla sp.* | pr |
| SM044 | Arthropoda | Insecta | Odonata | Cordulegastridae | *Cordulegaster sp.1* | pr |
| SM045 | Arthropoda | Insecta | Odonata | Cordulegastridae | *Cordulegaster sp.2* | pr |
| SM046 | Arthropoda | Insecta | Diptera | Chironomidae | *Cricotopus albiforceps* | sh |
| SM047 | Arthropoda | Insecta | Diptera | Ceratopogonidae | *Dasyhelea sp.* | pr |
| SM048 | Arthropoda | Insecta | Diptera | Chironomidae | *Diamesa sp.* | gc |
| SM049 | Arthropoda | Insecta | Coleptera | Gyrinidae | *Dineutus sp.1* | pr |
| SM050 | Arthropoda | Insecta | Coleptera | Gyrinidae | *Dineutus sp.2* | pr |
| SM051 | Arthropoda | Insecta | Trichoptera | Hydropsychidae | *Diplectrona sp.* | fc |
| SM052 | Arthropoda | Insecta | Diptera | Dixidae | *Dixa sp.* | fc |
| SM053 | Arthropoda | Insecta | Ephemeroptera | Ephemerellidae | *Drunella sp.* | gc |
| SM054 | Arthropoda | Insecta | Coleoptera | Elmidae | *Dubiraphia sp.1* | gc |
| SM055 | Arthropoda | Insecta | Coleoptera | Elmidae | *Dubiraphia sp.2* | gc |
| SM056 | Arthropoda | Insecta | Coleoptera | Dytiscidae | *Dytiscidae sp.1* | pr |
| SM057 | Arthropoda | Insecta | Coleoptera | Dytiscidae | *Dytiscidae sp.2* | pr |
| SM058 | Arthropoda | Insecta | Odonata | Coenagrionidae | *Enallagma sp.* | pr |
| SM059 | Arthropoda | Insecta | Ephemeroptera | Heptageniidae | *Epeorus melli* | sc |
| SM060 | Arthropoda | Insecta | Ephemeroptera | Heptageniidae | *Epeorus sp.* | sc |
| SM061 | Arthropoda | Insecta | Ephemeroptera | Ephemeridae | *Ephemera sp.* | gc |
| SM062 | Arthropoda | Insecta | Ephemeroptera | Ephemerellidae | *Ephemerella sp.1* | gc |
| SM063 | Arthropoda | Insecta | Ephemeroptera | Ephemerellidae | *Ephemerella sp.2* | gc |
| SM064 | Arthropoda | Insecta | Odonata | Corduliidae | *Epitheca sp.1* | pr |
| SM065 | Arthropoda | Insecta | Odonata | Corduliidae | *Epitheca sp.2* | pr |
| SM066 | Arthropoda | Insecta | Ephemeroptera | Ephemerellidae | *Eurylophella sp.* | gc |
| SM067 | Arthropoda | Malacostraca | Decapoda | Palaemonidae | *Exopalaemon modestus* | sh |
| SM068 | Arthropoda | Malacostraca | Decapoda | Palaemonidae | *Exopalaemon sp.1* | sh |
| SM069 | Arthropoda | Malacostraca | Decapoda | Palaemonidae | *Exopalaemon sp.2* | sh |
| SM070 | Arthropoda | Insecta | Hemiptera | Gerridae | *Gerris sp.* | pr |
| SM071 | Arthropoda | Insecta | Coleptera | Gyrinidae | *Gyrinus sp.* | pr |
| SM072 | Arthropoda | Insecta | Ephemeroptera | Leptophlebiidae | *Habrophlebia sp.1* | gc |
| SM073 | Arthropoda | Insecta | Ephemeroptera | Leptophlebiidae | *Habrophlebia sp.2* | gc |
| SM074 | Arthropoda | Insecta | [Trichoptera](https://en.wikipedia.org/wiki/Caddisfly) | Helicopsychidae | *Helicopsyche sp.* | sc |
| SM075 | Arthropoda | Insecta | Diptera | Empididae | *Hemerodromia sp.* | pr |
| SM076 | Arthropoda | Insecta | Ephemeroptera | Heptageniidae | *Heptagenia sp.* | sc |
| SM077 | Arthropoda | Insecta | Hemiptera | Corixidae | *Hesperocorixa sp.* | pr |
| SM078 | Arthropoda | Insecta | Ephemeroptera | Baetidae | *Heterocloeon sp.1* | sc |
| SM079 | Arthropoda | Insecta | Ephemeroptera | Baetidae | *Heterocloeon sp.2* | sc |
| SM080 | Arthropoda | Insecta | Trichoptera | Calamoceratidae | *Heteroplectron sp.* | gc |
| SM081 | Arthropoda | Insecta | Ephemeroptera | Ephemeridae | *Hexagenia sp.* | gc |
| SM082 | Arthropoda | Insecta | Diptera | Limoniidae | *Hexatoma sp.1* | pr |
| SM083 | Arthropoda | Insecta | Diptera | Limoniidae | *Hexatoma sp.2* | pr |
| SM084 | Arthropoda | Insecta | Diptera | Ephydridae | *Hydrellia sp.* | sh |
| SM085 | Arthropoda | Insecta | Trichoptera | Hydropsychidae | *Hydropsyche sp.* | fc |
| SM086 | Arthropoda | Arachnida | Trombidiformes | Hygrobatidae | *Hygrobates sp.* | pr |
| SM087 | Arthropoda | Insecta | Trichoptera | Limnephilidae | *Ironoquia sp.* | sh |
| SM088 | Arthropoda | Insecta | Odonata | Coenagrionidae | *Ischnura sp.* | pr |
| SM089 | Arthropoda | Insecta | Ephemeroptera | Isonychiidae | *Isonychia sp.* | fc |
| SM090 | Arthropoda | Malacostraca | Isopoda | Janiridae | *Jaera albifrons* | gc |
| SM091 | Arthropoda | Insecta | Odonata | Gomphidae | *Lanthus sp.1* | pr |
| SM092 | Arthropoda | Insecta | Odonata | Gomphidae | *Lanthus sp.2* | pr |
| SM093 | Arthropoda | Insecta | Trichoptera | Lepidostomatidae | *Lepidostoma sp.* | sh |
| SM094 | Arthropoda | Insecta | Ephemeroptera | Heptageniidae | *Leucrocuta sp.* | sc |
| SM095 | Arthropoda | Insecta | Odonata | Libellulidae | *Libellula sp.* | pr |
| SM096 | Arthropoda | Insecta | Ephemeroptera | Heptageniidae | *Maccaffertium sp.* | sc |
| SM097 | Arthropoda | Insecta | Coleoptera | Elmidae | *Macronychus glabratus* | gc |
| SM098 | Arthropoda | Insecta | Trichoptera | Hydropsychidae | *Macrostemum sp.* | fc |
| SM099 | Arthropoda | Insecta | Coleoptera | Elmidae | *Microcylloepus sp.1* | sc |
| SM100 | Arthropoda | Insecta | Coleoptera | Elmidae | *Microcylloepus sp.2* | sc |
| SM101 | Arthropoda | Insecta | Hemiptera | Micronectidae | *Micronecta sp.* | pr |
| SM102 | Arthropoda | Insecta | Hemiptera | Naucoridae | *Naucoridae sp.* | pr |
| SM103 | Arthropoda | Insecta | Ephemeroptera | Leptophlebiidae | *Neoleptophlebia sp.1* | gc |
| SM104 | Arthropoda | Insecta | Ephemeroptera | Leptophlebiidae | *Neoleptophlebia sp.2* | gc |
| SM105 | Arthropoda | Insecta | Hemiptera | Nepidae | *Nepa cinerea* | pr |
| SM106 | Arthropoda | Insecta | Trichoptera | Polycentropodidae | *Neureclipsis mandjurica* | fc |
| SM107 | Arthropoda | Insecta | Megaloptera | Corydalidae | *Nigronia sp.* | pr |
| SM108 | Arthropoda | Insecta | Diptera | Stratiomyidae | *Odontomyia sp.* | gc |
| SM109 | Arthropoda | Insecta | Odonata | Gomphidae | *Ophiogomphus sp.1* | pr |
| SM110 | Arthropoda | Insecta | Odonata | Gomphidae | *Ophiogomphus sp.2* | pr |
| SM111 | Arthropoda | Insecta | Diptera | Chironomidae | *Orthocladius sp.* | gc |
| SM112 | Arthropoda | Insecta | Plecoptera | Perlidae | *Paragnetina sp.* | pr |
| SM113 | Arthropoda | Insecta | Lepidoptera | Crambidae | *Parapoynx sp.* | sh |
| SM114 | Arthropoda | Insecta | Hemiptera | Naucoridae | *Pelocoris sp.* | pr |
| SM115 | Arthropoda | Insecta | Coleoptera | Haliplidae | *Peltodytes sp.* | sh |
| SM116 | Arthropoda | Insecta | Plecoptera | Perlidae | *Perlesta sp.* | pr |
| SM117 | Arthropoda | Insecta | Diptera | Ceratopogonidae | *Probezzia sp.* | pr |
| SM118 | Arthropoda | Insecta | Diptera | Chironomidae | *Procladius sp.* | pr |
| SM119 | Arthropoda | Insecta | Diptera | Simuliidae | *Prosimulium sp.* | fc |
| SM120 | Arthropoda | Insecta | Coleoptera | Psephenidae | *Psephenus sp.1* | sc |
| SM121 | Arthropoda | Insecta | Coleoptera | Psephenidae | *Psephenus sp.2* | sc |
| SM122 | Arthropoda | Insecta | Ephemeroptera | Baetidae | *Pseudocloeon sp.* | gc |
| SM123 | Arthropoda | Insecta | Diptera | Psychodidae | *Psychoda sp.* | gc |
| SM124 | Arthropoda | Insecta | Plecoptera | Pteronarcyidae | *Pteronarcys sp.* | sh |
| SM125 | Arthropoda | Insecta | Diptera | Ptychopteridae | *Ptychoptera sp.* | gc |
| SM126 | Arthropoda | Insecta | Hemiptera | Nepidae | *Ranatra Fabricius* | pr |
| SM127 | Arthropoda | Insecta | Diptera | Dolichopodidae | *Rhaphium sp.* | pr |
| SM128 | Arthropoda | Insecta | Ephemeroptera | Heptageniidae | *Rhithrogena sp.* | gc |
| SM129 | Arthropoda | Insecta | Trichoptera | Rhyacophilidae | *Rhyacophila sp.* | pr |
| SM130 | Arthropoda | Insecta | Coleoptera | Scirtidae | *Sacodes sp.* | sc |
| SM131 | Arthropoda | Insecta | Diptera | Ephydridae | *Scatella sp.* | sh |
| SM132 | Arthropoda | Insecta | Ephemeroptera | Ephemerellidae | *Serratella sp.* | gc |
| SM133 | Arthropoda | Insecta | Megaloptera | Sialidae | *Sialis sp.* | pr |
| SM134 | Arthropoda | Insecta | Diptera | Simuliidae | *Simulium sp.* | fc |
| SM135 | Arthropoda | Insecta | Diptera | Simuliidae | *Simulium sp.1* | fc |
| SM136 | Arthropoda | Insecta | Diptera | Simuliidae | *Simulium sp.2* | fc |
| SM137 | Arthropoda | Malacostraca | Decapoda | Potamidae | *Sinopotamon chekiangense* | sh |
| SM138 | Arthropoda | Malacostraca | Decapoda | Potamidae | *Sinopotamon sp.* | sh |
| SM139 | Arthropoda | Insecta | Ephemeroptera | Siphlonuridae | *Siphlonurus sp.* | gc |
| SM140 | Arthropoda | Insecta | Ephemeroptera | Metretopodidae | *Siphloplecton sp.* | gc |
| SM141 | Arthropoda | Insecta | Coleoptera | Elmidae | *Stenelmis sp.* | sc |
| SM142 | Arthropoda | Insecta | Diptera | Tabanidae | *Tabanidae sp.* | pr |
| SM143 | Arthropoda | Insecta | Diptera | Chironomidae | *Tanytarsus sp.* | pr |
| SM144 | Arthropoda | Insecta | Diptera | Chironomidae | *Thienemannimyia sp.* | pr |
| SM145 | Arthropoda | Insecta | Diptera | Tipulidae | *Tipula sp.1* | pr |
| SM146 | Arthropoda | Insecta | Diptera | Tipulidae | *Tipula sp.2* | pr |
| SM147 | Arthropoda | Insecta | Hemiptera | Gerridae | *Trepobates sp.* | pr |
| SM148 | Arthropoda | Insecta | Ephemeroptera | Leptohyphidae | *Tricorythodes sp.* | gc |
| SM149 | Arthropoda | Insecta | Coleoptera | Hydrophilidae | *Tropisternus sp.* | pr |
| SM150 | Mollusca | Gastropoda | Mesogastropoda | Viviparidae | *Angulyagra wilthelmin* | sc |
| SM151 | Mollusca | Gastropoda | Mesogastropoda | Viviparidae | *Bellamya aeruginosa* | sc |
| SM152 | Mollusca | Gastropoda | Mesogastropoda | Viviparidae | *Bellamya angularis* | sc |
| SM153 | Mollusca | Gastropoda | Mesogastropoda | Viviparidae | *Bellamya capillata* | sc |
| SM154 | Mollusca | Gastropoda | Mesogastropoda | Viviparidae | *Bellamya dispirariralis* | sc |
| SM155 | Mollusca | Gastropoda | Mesogastropoda | Viviparidae | *Bellamya heudei* | sc |
| SM156 | Mollusca | Gastropoda | Mesogastropoda | Viviparidae | *Bellamya lapidea* | sc |
| SM157 | Mollusca | Gastropoda | Mesogastropoda | Viviparidae | *Bellamya lapillorum* | sc |
| SM158 | Mollusca | Gastropoda | Mesogastropoda | Viviparidae | *Bellamya lithophaga* | sc |
| SM159 | Mollusca | Gastropoda | Mesogastropoda | Viviparidae | *Bellamya manhongensis* | sc |
| SM160 | Mollusca | Gastropoda | Mesogastropoda | Viviparidae | *Bellamya turritus* | sc |
| SM161 | Mollusca | Gastropoda | Littorinimorpha | Bithyniidae | *Bithynia fuchsiana* | sc |
| SM162 | Mollusca | Gastropoda | Mesogastropoda | [Bithyniidae](https://en.wikipedia.org/wiki/Bithyniidae) | *Bithynia leachi* | sc |
| SM163 | Mollusca | Gastropoda | Architaenioglossa | Viviparidae | *Bllamya quadrata* | sc |
| SM164 | Mollusca | Gastropoda | Sorbeoconcha | Pachychilidae | *Brotia herculea* | sc |
| SM165 | Mollusca | Gastropoda | Mesogastropoda | Viviparidae | *Cipangopaludina cathayensis* | sc |
| SM166 | Mollusca | Gastropoda | Mesogastropoda | Viviparidae | *Cipangopaludina chinensis* | sc |
| SM167 | Mollusca | Gastropoda | Mesogastropoda | Viviparidae | *Cipangopaludina fluminalis* | sc |
| SM168 | Mollusca | Gastropoda | Mesogastropoda | Viviparidae | *Cipangopaludina lecythoides* | sc |
| SM169 | Mollusca | Bivalvia | Venerida | Corbiculidae | *Corbicula fluminea* | fc |
| SM170 | Mollusca | Bivalvia | Venerida | Corbiculidae | *Corbicula largillierti* | fc |
| SM171 | Mollusca | Bivalvia | Venerida | Corbiculidae | *Corbicula nitens* | fc |
| SM172 | Mollusca | Gastropoda | Mesogastropoda | Planorbidae | *Gyraulus convexiusculus* | sc |
| SM173 | Mollusca | Gastropoda | Stylommatophora | Helicidae | *Helicigona heldreichi* | sc |
| SM174 | Mollusca | Gastropoda | Mesogastropoda | Planorbidae | *Hippeutis cantori* | sc |
| SM175 | Mollusca | Gastropoda | Mesogastropoda | Planorbidae | *Hippeutis umbilicalis* | sc |
| SM176 | Mollusca | Gastropoda | Mesogastropoda | Semisulcospiridae | *Hua aristarchorum* | sc |
| SM177 | Mollusca | Gastropoda | Mesogastropoda | Semisulcospiridae | *Hua luquanensis* | sc |
| SM178 | Mollusca | Gastropoda | Mesogastropoda | Semisulcospiridae | *Hua sp.* | sc |
| SM179 | Mollusca | Gastropoda | Mesogastropoda | Semisulcospiridae | *Koreoleptoxis davidi* | sc |
| SM180 | Mollusca | Gastropoda | Mesogastropoda | Semisulcospiridae | *Koreoleptoxis terminalis* | sc |
| SM181 | Mollusca | Bivalvia | Mytilida | [Mytilidae](https://en.wikipedia.org/wiki/Mytilidae) | *Limnoperna lacustris* | fc |
| SM182 | Mollusca | Gastropoda | Stylommatophora | Clausiliidae | *Miraphaedusa harryleei* | sc |
| SM183 | Mollusca | Gastropoda | Littorinimorpha | Bithyniidae | *Parafossarulus eximius* | sc |
| SM184 | Mollusca | Gastropoda | Mesogastropoda | Ampullariidae | *Pila tischberini* | sc |
| SM185 | Mollusca | Gastropoda | Mesogastropoda | Ampullariidae | *Pomacea archimedis* | sc |
| SM186 | Mollusca | Gastropoda | Mesogastropoda | Ampullariidae | *Pomacea canaliculata* | sc |
| SM187 | Mollusca | Bivalvia | Venerida | Veneridae | *Protothaca jedoensis* | fc |
| SM188 | Mollusca | Gastropoda | Mesogastropoda | Lymnaeidae | *Radix acuminata* | sc |
| SM189 | Mollusca | Gastropoda | Mesogastropoda | Lymnaeidae | *Radix auricularia* | sc |
| SM190 | Mollusca | Gastropoda | Mesogastropoda | Lymnaeidae | *Radix chlamys* | sc |
| SM191 | Mollusca | Gastropoda | Mesogastropoda | Lymnaeidae | *Radix hookeri* | sc |
| SM192 | Mollusca | Gastropoda | Mesogastropoda | Lymnaeidae | *Radix ovata* | sc |
| SM193 | Mollusca | Gastropoda | Mesogastropoda | Lymnaeidae | *Radix plicatula* | sc |
| SM194 | Mollusca | Gastropoda | Mesogastropoda | Lymnaeidae | *Radix rubiginosa* | sc |
| SM195 | Mollusca | Gastropoda | Mesogastropoda | Semisulcospiridae | *Semisulcospira cancellata* | sc |
| SM196 | Mollusca | Gastropoda | Mesogastropoda | Semisulcospiridae | *Semisulcospira jacquetiana* | sc |
| SM197 | Mollusca | Gastropoda | Mesogastropoda | Semisulcospiridae | *Semisulcospira libertina* | sc |
| SM198 | Mollusca | Gastropoda | Mesogastropoda | Semisulcospiridae | *Semisulcospira sp.* | sc |
| SM199 | Mollusca | Bivalvia | Veneroida | Corbiculidae | *Sphaerium lacustre* | fc |
| SM200 | Mollusca | Gastropoda | Mesogastropoda | Pachychilidae | *Sulcosira paludiformis* | sc |
| SM201 | Mollusca | Gastropoda | Mesogastropoda | Clausiliidae | *Synprosphyma ehrmanni* | sc |
| SM202 | Mollusca | Bivalvia | Mytilida | Mytilidae | *Vignadula atrata* | fc |
| SM203 | Mollusca | Gastropoda | Mesogastropoda | Viviparidae | *Viviparus chinensis chinensis* | sc |
| SM204 | Mollusca | Gastropoda | Mesogastropoda | Viviparidae | *Viviparus chinensis hainanensis* | sc |
| SM205 | Mollusca | Gastropoda | Mesogastropoda | Viviparidae | *Viviparus quadratus aeruginosus* | sc |
| SM206 | Mollusca | Gastropoda | Mesogastropoda | Viviparidae | *Viviparus quadratus ecarinatus* | sc |
| SM207 | Mollusca | Gastropoda | Mesogastropoda | Viviparidae | *Viviparus quadratus quadratus* | sc |
| SM208 | Mollusca | Gastropoda | Mesogastropoda | Viviparidae | *Viviparus quadratus reevei* | sc |
| SM209 | Mollusca | Gastropoda | Mesogastropoda | Viviparidae | *Viviparus wilhelmi* | sc |
| SM210 | Platyhelminthes | Turbellaria | Tricladida | Dugesiidae | *Dugesia sp.* | gc |
| SM211 | Platyhelminthes | Turbellaria | Tricladida | Planariidae | *Planaria sp.* | pr |

**Fig. S1** Results of agriculture, impervious, and forest for the low-disturbance and high-disturbance sites, respectively. Boxes show interquartile ranges (25th and 75th percentiles) and medians (middle lines); whiskers are non-outlier ranges beyond boxes, and dots are outliers. Asterisks indicate statistically significant differences between low- and high-disturbance sites (**P* < 0.05, ***P* < 0.01, and ****P* < 0.001).
